# Comprehensive genomic identification of cotton starch synthase genes reveals that *GhSS9* regulates drought tolerance

**DOI:** 10.3389/fpls.2023.1163041

**Published:** 2023-04-05

**Authors:** Maohua Dai, Xiaomin Yang, Quanjia Chen, Zhigang Bai

**Affiliations:** ^1^ Dryland Farming Institute, Hebei Academy of Agricultural and Forestry Sciences/Hebei Key Laboratory of Crops Drought Resistance, Hengshui, China; ^2^ Engineering Research Centre of Cotton, Ministry of Education/College of Agriculture, Xinjiang Agricultural University, Urumqi, China; ^3^ Cash Crop Research Institute of Jiangxi Province, Jiujiang, Jiangxi, China

**Keywords:** starch synthase, cotton, GhSS9, drought stress, gene network, VIGS

## Abstract

**Introduction:**

Starch metabolism is involved in the stress response. Starch synthase (SS) is the key enzyme in plant starch synthesis, which plays an indispensable role in the conversion of pyrophosphoric acid to starch. However, the SS gene family in cotton has not been comprehensively identified and systematically analyzed.

**Result:**

In our study, a total of 76 SS genes were identified from four cotton genomes and divided into five subfamilies through phylogenetic analysis. Genetic structure analysis proved that SS genes from the same subfamily had similar genetic structure and conserved sequences. A cis-element analysis of the SS gene promoter showed that it mainly contains light response elements, plant hormone response elements, and abiotic stress elements, which indicated that the SS gene played key roles not only in starch synthesis but also in abiotic stress response. Furthermore, we also conducted a gene interaction network for SS proteins. Silencing *GhSS9* expression decreased the resistance of cotton to drought stress. These findings suggested that SS genes could be related to drought stress in cotton, which provided theoretical support for further research on the regulation mechanism of SS genes on abiotic starch synthesis and sugar levels.

## Introduction

As the products of photosynthesis, plant starches are the main food for humans (Deschamps et al., 2008; Abdelgawad et al., 2020). Starches fall into two main categories. One kind of starch is made in the leaves of plants and temporarily stored as temporary starch, and the other is found in the fruits, seeds, and rhizomes of plants such as cereals and potatoes, which provide nutrients and energy for the development of offspring (Zeeman SC and Rees, 2010; Santelia and Zeeman, 2011). Both temporary starch and storage starch exist in the form of starch granules (Smith et al., 1997). Starches are glucose polymers connected by α-1,4 glycosidic and α-1,6 glycosidic bonds and are divided into amylose and amylopectin according to their structure (Gidley and Bociek, 1988; Buléon et al., 1998). Amylose has a small molecular weight and few α-1,6 glycosidic bond branches. Amylopectin has a higher polymerization degree, a higher molecular weight, and more α-1,6 glycosidic bond branches (Miles et al., 1985). Starch biosynthesis in maize seedlings contributes to the maintenance of leaf growth under drought stress and facilitates enhanced carbon acquisition upon recovery (Abdelgawad et al., 2020). Under drought stress, the transient starch in *Arabidopsis thaliana* is degraded to the carbon skeleton of sucrose and proline, or starches are broken down into soluble sugars that act as osmotic protectants to counteract osmotic pressure and oxidative damage (Zeeman et al., 2010; Zanella et al., 2016). The above pieces of evidence show that starches play an important role in plants’ resistance to drought stress.

Starches are synthesized with the participation of various enzymes related to starch synthesis. It is generally believed that the following key enzymes are required for starch synthesis: starch synthase (SS), ADP-glucose pyrophosphorylase (AGPase), starch branching enzyme (SBE), and starch debranching enzyme (DBE) (Dian et al., 2005). Among these enzymes, the SS gene family plays an important role in material storage and energy reserve (He et al., 2022). SSs can be divided into two categories according to their degree of binding to starch granules, enzymatic characteristics, and gene structure. One is granule-bound starch synthase (GBSS), and the other is soluble starch synthase (SS) (Dian et al., 2005). Dian et al. identified that amylose in rice leaves was synthesized by GBSS II (Dian et al., 2003; Zhang et al., 2021). GBSS II was also isolated from the non-storage organs of pea and wheat, which were used in the synthesis of temporary starch (Denyer et al., 1993; Yasunori, 2002). Soluble starch synthase can be divided into SSI, SSII, SSIII, and SSIV according to its amino acid structure (Mu-Forster, 1996). The activity ratios of various starch synthases in different plants or different tissues of the same plant are different (Smith et al., 1997).

The level of sugar in higher plants regulates the whole growth and development process from germination to flowering to senescence (Ding, 1998). Sugar is not only used for energy metabolism in plants but also plays an important role in plant growth and development, metabolic regulation, and stress resistance (Zhao, 2006). Glucose 1-phosphate is produced by glucose-phosphorylase and converted to starch by starch synthase. Inhibiting starch synthesis can result in pollen abortion, organ atrophy, and delayed development or aging (Dorion and Saini, 1996). The increase in sugar levels in plants will promote the synthesis and accumulation of starch (Matt et al., 1998). Starch is not only a storage compound but also a regulator under stress conditions. When carbohydrate assimilation is impaired under stress, starch metabolism can buffer the adverse effects of stress-induced carbon depletion (Kaplan and Guy, 2004; Wim et al., 2014). Under drought stress, the starch in broad bean leaves was depleted, but it accumulated in the pods (Hernández et al., 2012). The activity of SS family genes and the accumulation rate of starch decreased in wheat under drought stress (Hou et al., 2017).

Based on the role of SSs in starch synthesis, SS genes may be a good target for crop improvement and abiotic stress resistance. As an important cash crop, cotton’s growth and development are affected by biological and abiotic factors. Otherwise, the SS family in cotton has not been studied. In our study, we used bioinformatics to synthesize the whole genomes of the SS families of *G. arboreum*, *G. raimondii*, *G. hirsutum*, and *G. barbadense*. Our results lay the foundation for further research into the mechanism by which SS genes regulate starch synthesis and sugar levels during plant development and their response to abiotic stress.

## Materials and methods

### Identification of SS family in cotton

To obtain SS family members in cotton, we downloaded the reference sequence file of *A. thaliana* from The Arabidopsis Information Resource online database (TAIR 10.1) (https://www.arabidopsis.org/). The newly updated version of the four cotton genome files, *G. arboretum* (BGI), *G. raimondii* (BGI), *G. hirsutum* (ZJU), and *G. barbadense* (ZJU), was downloaded from COTTONGEN (https://www.cottongen.org/). The reference sequence file of *A. thaliana* SSs was used as a query target to search against the genome file of four cotton species using local software Blast 2.13. Thus, candidate gene members of the SS family in four cotton genomes were obtained. The Hidden Markov Model (HMM) profile of PF08323 was downloaded from Pfam (https://pfam.xfam.org/). These genes were further screened using Pfam (https://pfam.xfam.org/) and SMART (http://SMART.emblheidelberg.de/). We only retained genes from the Glyco_transf_5 domain. We also analyzed theoretical isoelectric point (pI), molecular weight (MW), and subcellular location predictions for these SS proteins. We used several web sites, such as Cell-PLoc 2.0 (http://www.csbio.sjtu.edu.cn/bioinf/plant-multi/), to predict the subcellular location of the SSs. Expasy (https://web.expasy.org/compute_pi/) predicted the MW and pI of SSs.

### Phylogenetic analysis

We aligned the amino acid sequences of *A. thaliana* and *Oryza sativa* L. and four cotton species by ClustalX v1.83 (Larkin et al., 2007) with default parameters. We used MEGA 7.0 (Kumar, 2016) to find the best model and build the developmental tree. The SS protein sequences of the four cotton genomes were entered into MEGA 7.0 software. Muscle was used for multiple sequence alignment, and the neighbor method was used to construct the intra-species evolutionary tree.

### Analysis of the conserved motifs and gene structure of SS genes

We used the MEME (http://meme-suite.org/) website to predict the conserved motif of the SS proteins. The GFF files of the four genomes were merged using cmd instructions. Figures of the SS phylogenetic tree, conserved motifs, and introns and exons were drawn with TBtools software using nwk profiles (Chen et al., 2018), MAST profiles, and GFF profiles.

### Chromosomal location analysis

Download the gene annotation files (GFF) for the four cotton genomes from COTTONGENE (https://www.cottongen.org/). The genes displayed on the chromosome were obtained by TBtools software using the GFF file.

### Collinearity analysis

To investigate the collinearity of SS genes in four cotton genomes, we used MCScanX (Wang et al., 2012) software to analyze the synchronous relationships between duplicate gene pairs in four cotton genomes. Graphical results were displayed by TBtools software (Chen et al., 2018).

### Calculation of Ka/Ks

The cds sequences of SS genes from four cotton genomes were downloaded from COTTONGENE. We calculated the nonsynonymous (Ka) and synonymous (Ks) substitution rates and Ka/Ks ratio with the KaKs Calculator 2.0 program using the homologous gene pairs of four cotton genomes obtained during collinearity analysis (Dapeng et al., 2010).

### Analysis of the cis−elements of SS genes

We used TBtools software to obtain 1,000-bp DNA sequences upstream of SS genes in four cotton genomes. The cis element in the promoter was predicted by the PlantCARE website (http://bioinformatics.psb.ugent.be/webtools/plantcare/html/). We selected cis elements that respond to plant hormones, light, and other stresses for further analysis.

### Interaction network of GhSS proteins

To analyze the interaction networks of GhSS proteins, we performed this analysis using the STRING database (https://STRING-db.org/).

### Virus−induced gene silencing and drought treatment

A total of 403 bp of *GhSS9* was inserted into the pYL156 vector (which was cut with the restriction enzymes XbaI and BamHI). We constructed pYL156:GhSS9, the positive control pYL156:PDS, and the negative control pYL156. The primers for the *GhSS9* silencing fragment were as follows: the forward primer, ‘5-GTGAGTAAGGTTACCGAATTCTATTATCTTTGTGGGAGCTGAGGTT-3’ and the reverse primer, ‘5-CGTGAGCTCGGTACCGGATCCTTGCTGCTATTTAAATTCAGAACTCTT-3.’ When plants reached the three-leaf stage, the control group was irrigated with pure water as required, while the experimental group was controlled in soil drought stress by no watering. After 3 days of treatment, we collected the true leaves of the plants to analyze the relative expression level of *GhSS9*.

## Results

### Identification of SS genes

We identified 12, 14, 25, and 25 SS members from *G. arboreum*, *G. raimondii*, *G. hirsutum*, and *G. barbadense*. According to the position of the gene on the chromosome, we renamed 76 SS members. We further analyzed SS family members’ length, molecular weight, theoretical isoelectric point, and subcellular localization prediction. The length of the SS protein sequence was different in cotton, but the physicochemical properties were similar. All 76 genes encode proteins ranging from 177 (*GhSS16*) to 1,185 (*GaSS5*) amino acids, with pIs varying from 4.25 (*GbSS21*) to 8.62 (*GaSS3*) and MWs varying from 135.17 (*GaSS5*) kDa to 19.013 (*GhSS16*) kDa (**Table 1**). For the prediction of the subcellular localization of SS proteins, we found that most of the SS proteins were localized to chloroplasts, and only 12 proteins were localized to the extracellular domain.

**Table 1:** Information of the SS genes in cotton.

Gene name	Locus ID	Chromosome Position	Transcript Length (bp)	Gene Length (bp)	Protein Length (aa)	Molecular Weight (kDa)	Isoelectric Point	Subcellular Prediction
GaSS1	Cotton_A_05932_BGI-A2_v1.0	Chr1:140573723..140578374+	4651	2196	731	81.227	5.46	Chloroplast
GaSS2	Cotton_A_29910_BGI-A2_v1.0	Chr2:86130759..86139632+	8873	2016	671	75.647	6.77	Chloroplast
GaSS3	Cotton_A_34955_BGI-A2_v1.0	Chr5:33843588..33846626+	3038	1830	609	67.111	8.62	Chloroplast
GaSS4	Cotton_A_13194_BGI-A2_v1.0	Chr6:15698849..15701607+	2758	1827	608	67.084	6.34	Chloroplast
GaSS5	Cotton_A_25631_BGI-A2_v1.0	Chr9:74934872..74940781+	5909	3558	1185	135.17	6.25	Extracellular
GaSS6	Cotton_A_17559_BGI-A2_v1.0	Chr9:89705935..89712616+	6681	3135	1044	118.228	5.77	Chloroplast
GaSS7	Cotton_A_04683_BGI-A2_v1.0	Chr10:20189943..20198403-	8460	3489	1162	132.724	6.24	Extracellular
GaSS8	Cotton_A_22105_BGI-A2_v1.0	Chr11:26022930..26026865+	3935	2253	750	82.949	5.74	Chloroplast
GaSS9	Cotton_A_08092_BGI-A2_v1.0	Chr11:62987706..62992597+	4891	1971	656	72.697	5.05	Chloroplast
GaSS10	Cotton_A_08125_BGI-A2_v1.0	Chr11:63240798..63243999-	3201	1827	608	66.958	8.47	Chloroplast
GaSS11	Cotton_A_05299_BGI-A2_v1.0	Chr12:48675283..48683515+	8227	3159	1052	119.237	5.63	Chloroplast
GaSS12	Cotton_A_20346_BGI-A2_v1.0	Chr12:137175811..137182383+	6572	1944	647	71.91778	6.05	Chloroplast
GrSS1	Cotton_D_gene_10013308	Chr01:5598457…5603107+	4651	2196	731	81.117	5.769	Chloroplast
GrSS2	Cotton_D_gene_10024047	Chr01:23646928…23654622+	7695	3024	1007	114.091	5.594	Chloroplast
GrSS3	Cotton_D_gene_10022657	Chr04:10268874…10272618+	3745	2531	609	66.988	8.5	Chloroplast
GrSS4	Cotton_D_gene_10012591	Chr06:43056052…43061045+	4994	1971	656	72.782	4.867	Chloroplast
GrSS5	Cotton_D_gene_10015367	Chr06:43293981…43297188+	3208	1821	606	66.652	8.147	Chloroplast
GrSS6	Cotton_D_gene_10016794	Chr08:7142124…7144888+	2765	1650	549	60.557	7.256	Chloroplast
GrSS7	Cotton_D_gene_10033842	Chr09:21343492…21351966+	8475	3489	1162	132.827	6.724	Extracellular
GrSS8	Cotton_D_gene_10040942	Chr10:23975409…23977272-	1864	570	189	20.253	8.089	Chloroplast
GrSS9	Cotton_D_gene_10031225	Chr11:985740…992232-	6493	4149	1153	131.428	6.475	Extracellular
GrSS10	Cotton_D_gene_10031504	Chr11:3185920…3192480-	6561	2889	962	108.615	6.104	Chloroplast
GrSS11	Cotton_D_gene_10006008	scaffold290:154678…158515+	3838	2175	724	80.357	5.035	Chloroplast
GrSS12	Cotton_D_gene_10008486	scaffold285:712164…718641+	6478	1944	647	72.072	6.42	Chloroplast
GrSS13	Cotton_D_gene_10010688	scaffold222:75924…76829+	906	906	301	34.062	5.785	Chloroplast
GrSS14	Cotton_D_gene_10013359	scaffold180:848473…857423+	8951	1926	641	71.983	7.082	Chloroplast
GbSS1	GB_A02G1761	A02:98299054…98307933+	8880	2073	690	77.701	7.126	Chloroplast
GbSS2	GB_A05G1732	A05:16661154…16669615+	8462	3489	1162	132.725	6.597	Extracellular
GbSS3	GB_A05G4447	A05:107379972…107386470+	6499	1944	647	71.992	6.331	Chloroplast
GbSS4	GB_A06G2286	A06:117132110…117140342-	8233	3159	1052	119.256	5.725	Chloroplast
GbSS5	GB_A07G2420	A07:90943188…90947677+	4490	2040	679	75.098	5.581	Chloroplast
GbSS6	GB_A08G1355	A08:79330426…79333473-	3048	1830	609	67.172	8.497	Chloroplast
GbSS7	GB_A09G0181	A09:3698540…3702608-	4069	2253	750	82.988	5.917	Chloroplast
GbSS8	GB_A09G2111	A09:73121959…73130900+	8942	3102	1033	115.461	6.162	Chloroplast
GbSS9	GB_A09G2142	A09:73365067…73368271+	3205	1827	608	66.921	8.187	Chloroplast
GbSS10	GB_A10G0124	A10:1075690…1081600-	5911	3462	1153	131.275	6.744	Extracellular
GbSS11	GB_A10G0399	A10:3456087…3462770-	6684	3135	1044	118.172	6.049	Chloroplast
GbSS12	GB_A12G2707	A12:98508732…98511629-	2898	1740	579	64.236	7.275	Chloroplast
GbSS13	GB_D03G0326	D03:3519642…3528626-	8985	2073	690	77.544	7.105	Chloroplast
GbSS14	GB_D04G0016	D04:151693…158435-	6743	1944	647	72.102	6.42	Chloroplast
GbSS15	GB_D05G1757	D05:15059306…15067748+	8443	3489	1162	132.827	6.767	Extracellular
GbSS16	GB_D06G1349	D06:31157612…31162477+	4866	1017	338	37.808	5.412	Chloroplast
GbSS17	GB_D06G2386	D06:61740366…61751025-	10660	3252	1083	122.672	6.002	Chloroplast
GbSS18	GB_D07G2390	D07:52912370…52916947+	4578	2196	731	81.379	5.679	Chloroplast
GbSS19	GB_D08G1322	D08:39182218…39185261+	3044	1830	609	67.016	8.5	Chloroplast
GbSS20	GB_D09G0157	D09:3475019…3478932-	3914	2259	752	83.461	5.576	Chloroplast
GbSS21	GB_D09G1958	D09:48280845…48285821+	4977	1971	656	72.639	4.825	Chloroplast
GbSS22	GB_D09G1991	D09:48495518…48498719+	3202	1827	608	66.653	8.191	Chloroplast
GbSS23	GB_D10G0125	D10:1021366…1027189-	5824	3462	1153	131.208	6.455	Extracellular
GbSS24	GB_D10G0404	D10:3344628…3356225-	11598	3120	1039	117.711	6	Chloroplast
GbSS25	GB_D12G2710	D12:57707314…57710086-	2773	1827	608	67.068	6.381	Chloroplast
GhSS1	GH_A02G1732.1	A02:104491293…104500171+	8879	2073	690	77.671	7.239	Chloroplast
GhSS2	GH_A05G1711.1	A05:16206432…16214893+	8462	3489	1162	132.725	6.597	Extracellular
GhSS3	GH_A05G4356.1	A05:110561301…110567799+	6499	1947	648	72.091	6.331	Chloroplast
GhSS4	GH_A06G2253.1	A06:125423524…125431717-	8194	3159	1052	119.24	5.746	Chloroplast
GhSS5	GH_A07G2327.1	A07:92499849…92504493+	4645	2196	731	81.277	5.685	Chloroplast
GhSS6	GH_A08G1286.1	A08:83900643…83903690-	3048	1830	609	67.146	8.497	Chloroplast
GhSS7	GH_A09G0154.1	A09:3598910…3602625-	3716	2055	684	76.408	6.216	Chloroplast
GhSS8	GH_A09G1998.1	A09:77010191…77015075+	4885	1971	656	72.73	4.828	Chloroplast
GhSS9	GH_A09G2029.1	A09:77250958…77254164+	3207	1827	608	66.87	8.187	Chloroplast
GhSS10	GH_A10G0118.1	A10:937473…943383-	5911	3462	1153	131.319	6.744	Extracellular
GhSS11	GH_A10G0401.1	A10:3394242…3400925-	6684	3135	1044	118.136	6.212	Chloroplast
GhSS12	GH_A12G2609.1	A12:104424370…104427129-	2760	1827	608	67.14	6.923	Chloroplast
GhSS13	GH_D03G0332.1	D03:3500078…3509060-	8983	2073	690	77.47	7.105	Chloroplast
GhSS14	GH_D04G0013.1	D04:237881…244435-	6555	1947	648	72.171	6.42	Chloroplast
GhSS15	GH_D05G1743.1	D05:14809632…14818068+	8437	3489	1162	132.669	6.723	Extracellular
GhSS16	GH_D06G1301.1	D06:31949898…31952341+	2444	534	177	19.013	8.48	Chloroplast
GhSS17	GH_D06G2293.1	D06:64282074…64290270-	8197	3159	1052	119.233	5.879	Chloroplast
GhSS18	GH_D07G2271.1	D07:54299057…54303634+	4578	2196	731	81.351	5.586	Chloroplast
GhSS19	GH_D08G1263.1	D08:40766146…40769189+	3044	1830	609	67.033	8.449	Chloroplast
GhSS20	GH_D09G0162.1	D09:3533864…3537785-	3922	2265	754	83.65	5.379	Chloroplast
GhSS21	GH_D09G1945.1	D09:46599042…46604020+	4979	1971	656	72.687	4.861	Chloroplast
GhSS22	GH_D09G1976.1	D09:46819582…46822783+	3202	1827	608	66.653	8.191	Chloroplast
GhSS23	GH_D10G0128.1	D10:1045921…1051744-	5824	3462	1153	131.131	6.474	Extracellular
GhSS24	GH_D10G0421.1	D10:3351239…3362836-	11598	3120	1039	117.711	6	Chloroplast
GhSS25	GH_D12G2632.1	D12:58611668…58614429-	2762	1827	608	67.095	6.641	Chloroplast

### Phylogenetic analysis of the SS family

**Figure 1:**
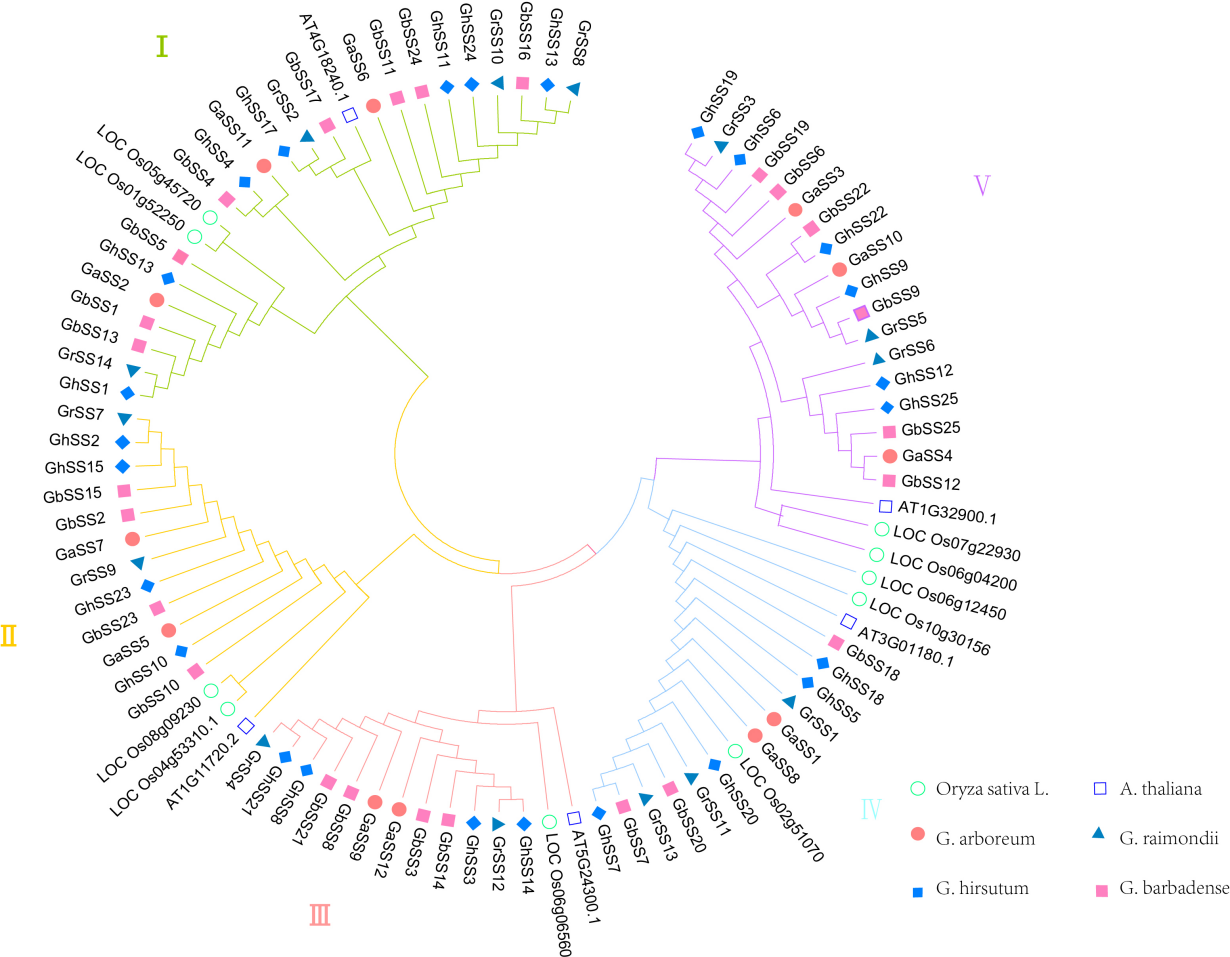
Phylogenetic analysis of SS protein from *Oryza sativa* L., *Arabidopsis thaliana*, and cotton.

In order to study the evolutionary relationships of the SS family genes in *O. sativa* L., *A. thaliana*, and cotton, we constructed phylogenetic trees using protein sequences of SS family members (**Figure 1**). The results showed that the SS family was divided into five subfamilies; each subfamily had 25, 16, 14, 16, and 20 members in cotton, respectively. The total number of *G. hirsutum* (AD_1_) and *G. barbadense* (AD_2_) SS was the same in each subfamily. The total number of SS members of *G. arboreum* (A) and *G. raimondii* (D) was the same as the number of SS members of allotetraploid cotton (*G. hirsutum* (AD_1_) or *G. barbadense* (AD_2_) in the I, II, III, and V subfamilies. This was consistent with the hypothesis of the origin and history of allotetraploid cotton (Brubaker et al., 1999). The I subfamily has 25 members, making it the largest subfamily. We speculate that the I subfamily member may play an active role in starch synthesis in cotton and *A. thaliana*.

### Structure of SS genes and conserved motifs

**Figure 2:**
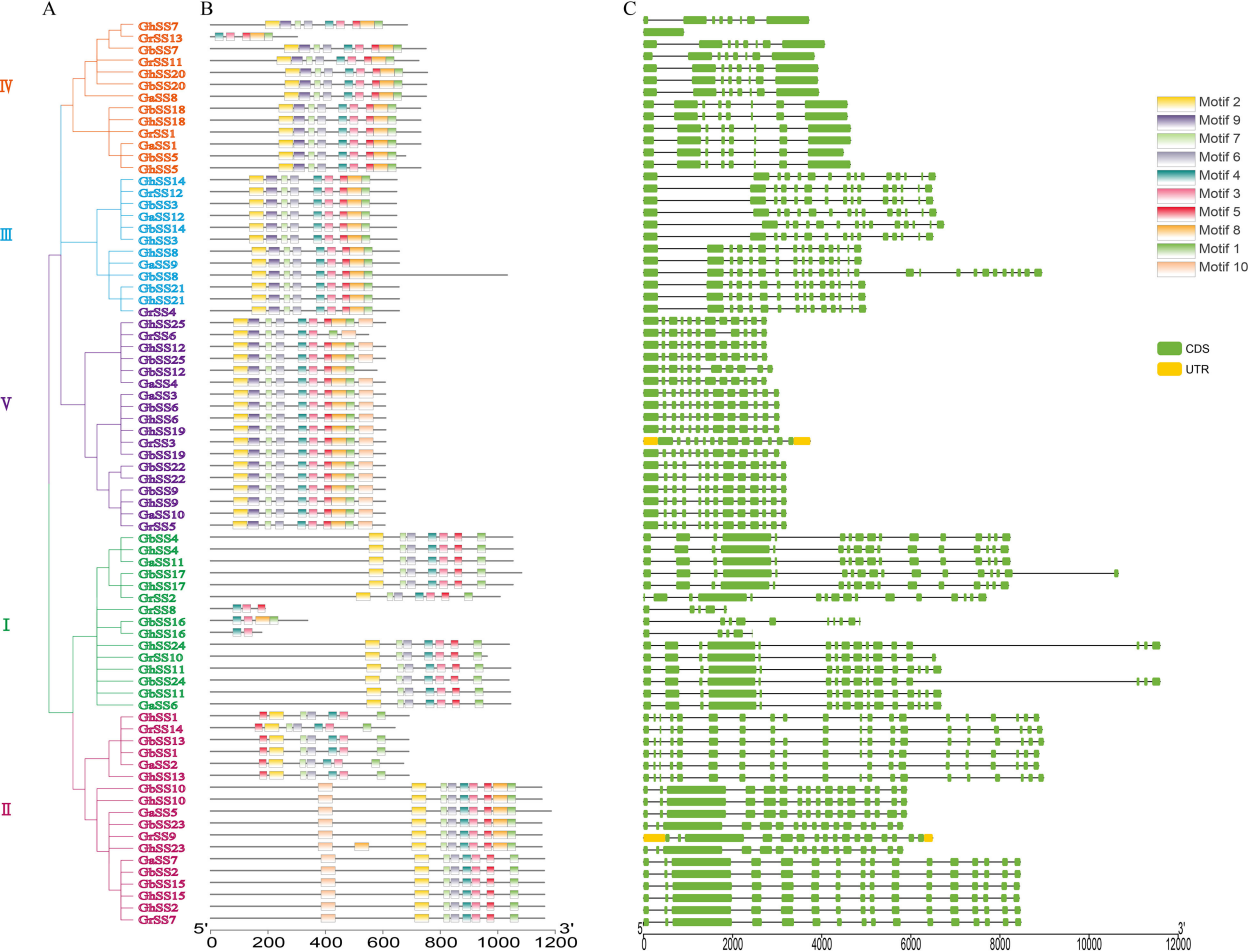
Conserved motifs and exon-intron structure of SS genes in *G arboreum*, *G raimondii*, *G hirsutum*, and *G barbadense*. **(A)** Phylogenetic tree of SS genes. **(B)** Conserved motifs of SS proteins. **(C)** The exon–intron structure of SS genes.

We analyzed the exon-intron structure and conserved motifs of the SS genes as shown in **Figure 2**. Ten motifs (1–10) were defined in SS members using MEME. All SS proteins contained motifs 3 and 4. We inferred that motifs 3 and 4 were important components of SS proteins. Genes in the same subfamily had similar gene structures and conserved motifs, and genes were specific between different subfamilies, indicating that SS families were more conserved during evolution and played multiple functions (Xwa et al., 2020). For example, subfamily III contained motifs 1–9. The members of subfamily V all contained motifs 1–4, 6–9. The number of exons in each SS family varied from 1 to 26. The number of exons was different in different subfamilies, and most genes in the same subfamily had the same number of exons.

### Chromosomal distribution

**Figure 3:**
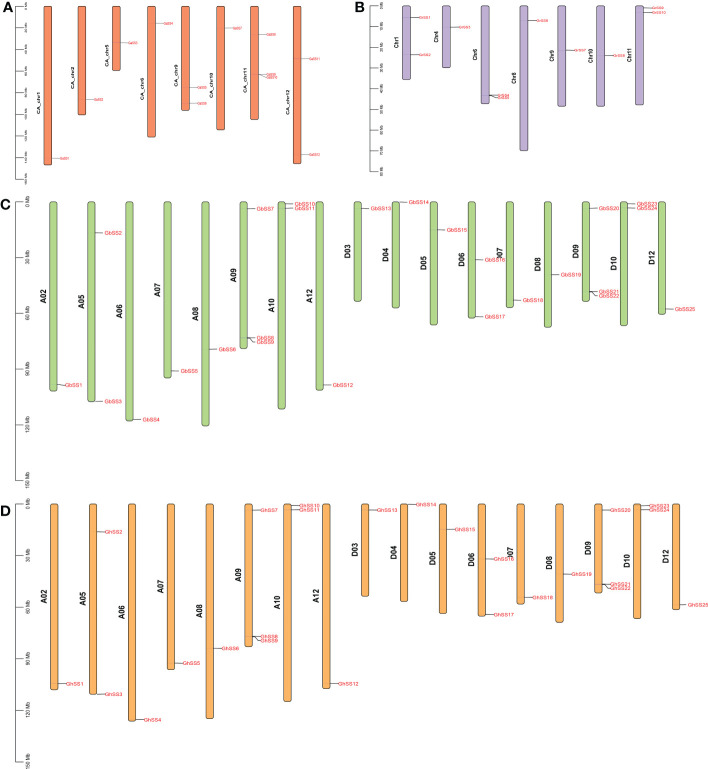
Chromosomal locations of SS genes in G arboreum, G raimondii, G hirsutum, and G barbadense. **(A)** G arboretum, **(B)** G raimondii, **(C)** G barbadense, and **(D)** G hirsutum.

To further study the chromosomal distribution and inheritance of SS family members, we mapped all SS genes to the corresponding chromosomes. As shown in **Figure 3**, 72 of 76 SS genes were mapped to chromosomes. For the GaSS genes from *G. arboreum*, 12 GaSSs were located on eight chromosomes (CA_chr1, CA_chr2, CA_chr5, CA_chr6, CA_chr9, CA_chr10, CA_chr11, and CA_chr12). For the GrSS genes from *G. raimondii*, 10 of 14 GrSSs were mapped to seven chromosomes (Chr1, Chr4, Chr6, Chr8, Chr9, Chr10, and Chr11), and four GrSSs were mapped to scaffolds. For the GhSS and GbSS gene families, they shared a similar chromosomal distribution pattern, with 25 genes assigned to chromosome 17, respectively. Twelve genes were assigned to eight chromosomes (A02, A05, A06, A07, A08, A09, A10, and A12) in group A, and 13 genes to nine chromosomes (D03, D04, D05, D06, D07, D08, D09, D10, and D12) in group D.

### Collinearity analysis

**Figure 4:**
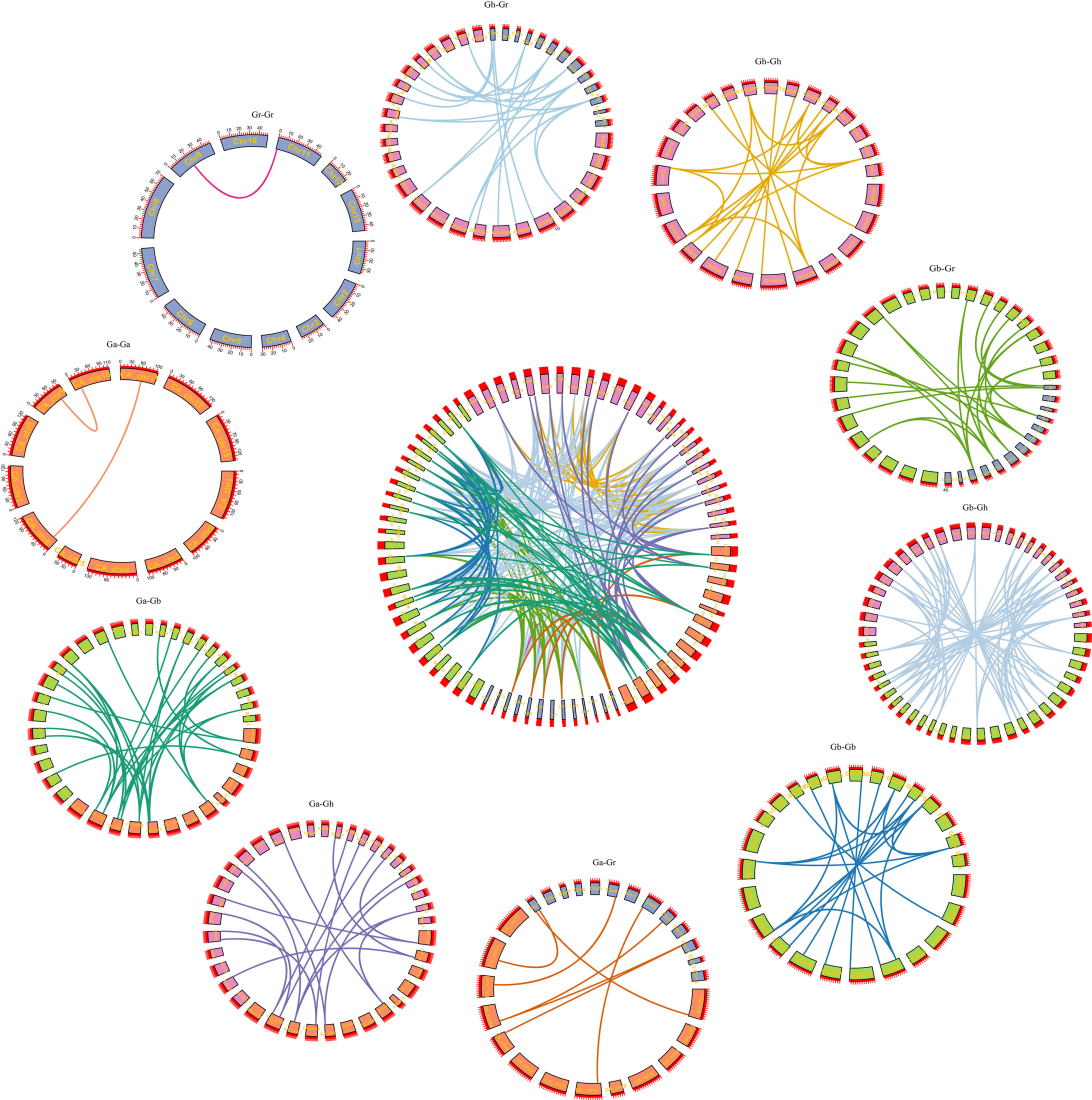
Syntenic relationship of SS duplicate gene pairs in cotton.

Through homology analysis of SS genes in *G. arboreum*, *G. raimondii*, *G. hirsutum*, and *G. barbadense*, we mapped the relationship between SS genes from the four cotton varieties (**Figure 4**). By comparing the genomes of Ga-Ga, Ga-Gb, Ga-Gh, Gb-Gb, Gb-Gr, Gb-Gh, Gr-Gh, Gr-Gh, Gr-Gr, and Gh-Gh, we identified a total of 217 linear/similar gene pairs. Among them, 45 duplicate genes were cloned into fragments, and 172 duplicate genes were cloned into the whole genome. Among them, Ga-Ga, Gb-Gb, Gh-Gh, and Gr-Gr had 2, 21, 21 and 1 pair of co-linear gene segments, respectively. Ga-Gh, Ga-Gb, Ga-Gr, Gb-Gh, Gb-Gr, and Gh-Gr replicated 33, 19, 9, 57, 27, and 27 linear/similar gene pairs, respectively. Therefore, we conjecture that the main driving force behind the evolution of SS family genes is genome-wide replication, followed by fragment replication.

### Selection pressure analysis

**Figure 5:**
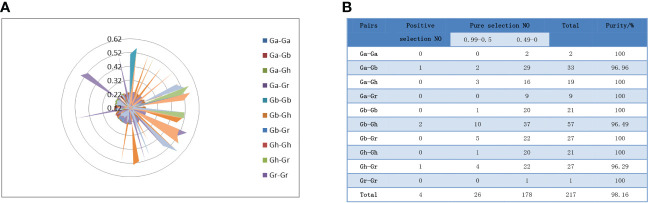
Analysis of the non-synonymous (Ka) to synonymous (Ks) ratio. **(A)** Nonsynonymous (Ka) and synonymous (Ks) divergence values for Ga–Ga, Ga-Gb, Ga-Gr, Ga-Gh, Gb-Gb, Gb-Gr, Gb-Gh, Gr-Gr, Gr-Gh, and Gh-Gh are shown in the circular chart. **(B)** Prediction number of the duplicate gene pairs involved in different combinations of four cotton species.

To study the phylogeny of SS gene pairs, we performed selective stress analysis. Ratios of nonsynonymous substitution rate (Ka) and synonymous substitution rate (Ks) were calculated for 217 gene pairs (**Figure 5A**). The results showed that the Ka/Ks values of 213 gene pairs were less than 1, the Ka/Ks values of 178 genes were between 0 and 0.5, and the Ka/Ks values of 26 genes were between 0.5 and 0.99 (**Figure 5B**). That is to say, these genes had a negative selection effect, which indicated that they had experienced purification selection pressure after gene duplication events. Since the Ka/Ks ratios of GbSS23-GhSS23, GbSS8-GhSS8, GrSS2-GhSS17, and GbSS8-GaSS9 were greater than 1, it was considered that these genes had positive selection effects in the process of evolution.

### Promoters and conservative domain analysis of SS genes

**Figure 6:**
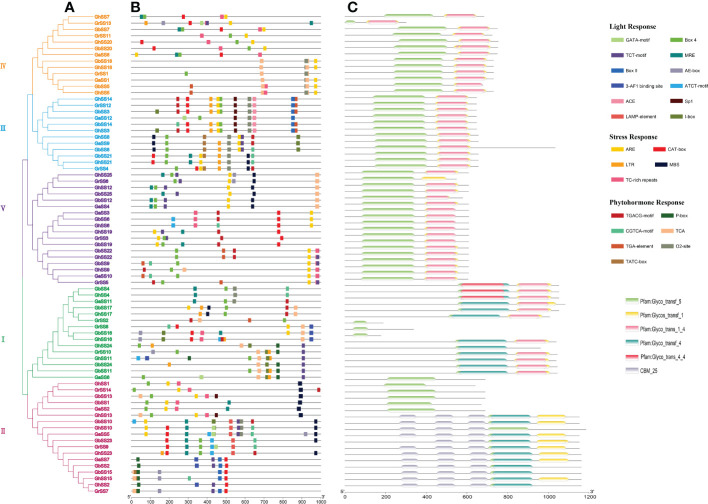
Promoters and conservative domains of SS genes in *G arboreum*, *G raimondii*, *G hirsutum*, and *G barbadense*. **(A)** Phylogenetic tree of SS genes. **(B)** Promoters of SS proteins. **(C)** The conservative domain of SS genes.

To better investigate the mechanisms of gene regulation, we utilized PlantCARE (**Figure 6B**) to identify several cis-regulatory elements in the promoter regions of each SS gene, which could be divided into three categories. The first was the light response element, which include Box 4, TCT-motif, MRE, i-Box, Box II, ae-Box, ATCT-motif, Sp1,3-AF1 binding site, GATA-motif, LAMP-element, Ace, for a total of 12 elements, which were located upstream of 51, 40, 25, 16, 11, 10, 9, 8, 7, 7, and 6 genes, respectively. The second type was the stress response element, including ARE, CAT-box, MBS, TC-rich repeats, and LTR, with a total of five elements located upstream of 53, 34, 28, 26, and 21 genes, respectively. The third class was the phytohormone response element, including TCA, TGACG-motif, CGTCA-motif, O2-site, TGA-element, P-box, TATC-box, with a total of seven elements, which were located upstream of 71, 38, 35, 24, 13, 12, and 8 genes, respectively.

To study the protein domains of SS genes, we used HMMER (https://www.ebi.ac.uk/tools/HMMER/search/phmmer) to analyze the conserved domains of SS genes. As shown in **Figure 6C**, each gene contained a Glyco_transf_5 domain. Different subfamilies had different domains, but the same subfamily had similar domains. Subfamily I genes all contained Glyco_transf_5, Glycos_transf_1, Glyco_trans_1_4 and Glyco_transf_4 domains except for *GrSS8*, *GbSS16*, and *GhSS16* genes. Subfamily II contains two types of domains. One type only contained the Glyco_transf_5 domain, however, the other type contained the Glyco_transf_5, Glycos_transf_1 and Glyco_trans_4 domains. Subfamily III, IV, and V genes all contained Glyco_transf_5, Glycos_transf_1 and Glyco_trans_1_4 domains, except for *GrSS6* (**Figure 6A**).

### Interaction network of GhSS proteins

**Figure 7:**
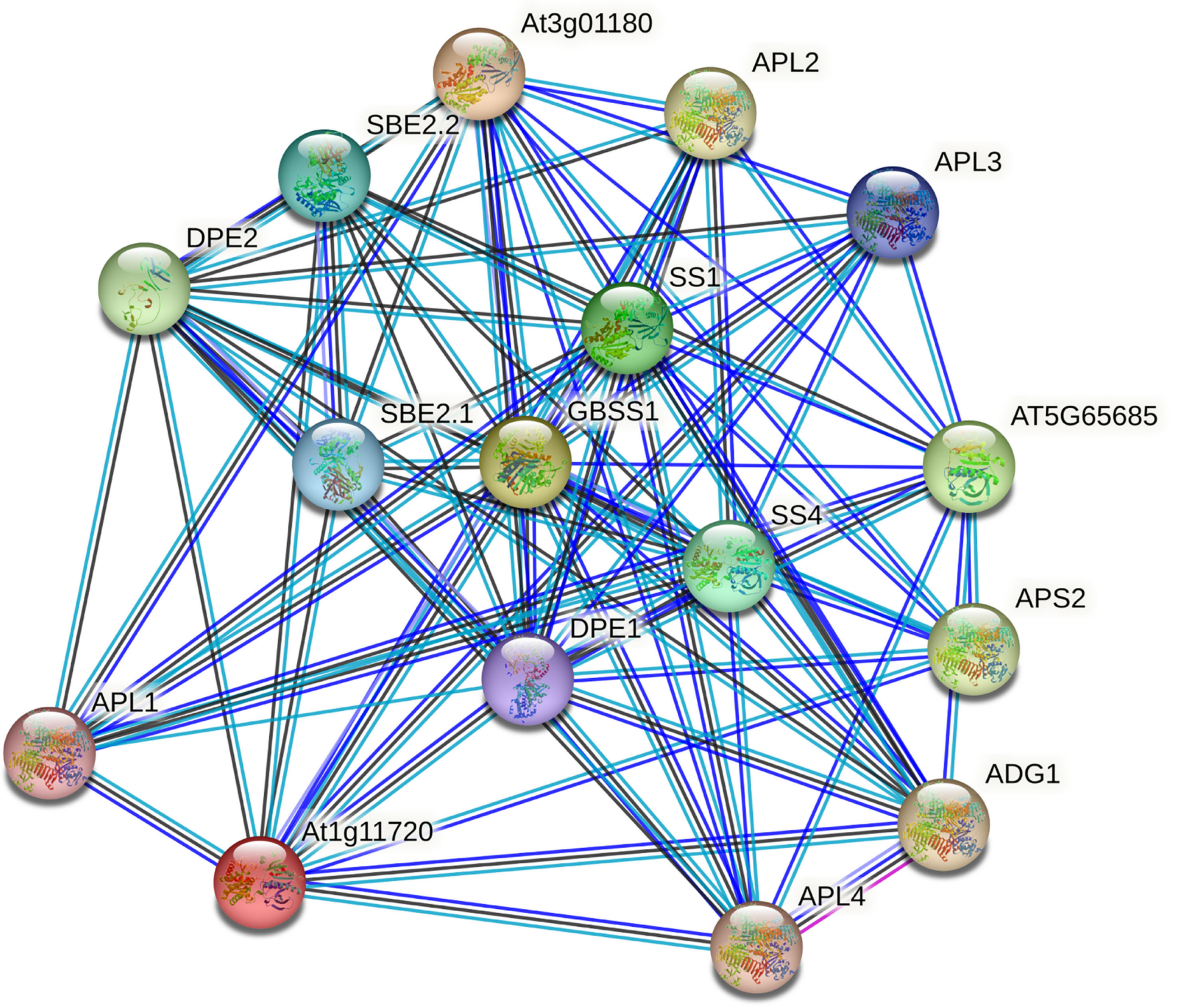
Interaction network of GhSS proteins.

To further investigate the function of the GhSS protein, we used STRING data (https://STRING-db.org/) for interaction network analysis. We compared GhSS proteins with *A. thaliana* proteins to obtain *A. thaliana* homologs and searched for them using multiple sequences (**Figure 7**). Results showed that sugar levels not only regulate gene expression, metabolism, growth in bacteria, yeast, and animals but also influence signal cell growth and development. In vascular plants, it also serves as a signal regulating multiple metabolic pathways and development processes. There are two main processes in the protein–protein interaction network. One was the synthesis of starch, and the other was the degradation of starch. SS family (GBSS1, SS1, and SS2), GlgB subfamily genes (EMB2729 (1,4-alpha-glucan-branching enzyme), SBE (1,4-alpha-glucan-branching enzyme), APL (glucose-1-phosphate adenylyltransferase large subunit), and SPL genes were involved in starch synthesis. The SS family gene DPE2(4-alpha-glucanotransferase) is involved in starch hydrolysis (Takaha et al., 1993). We hypothesized that cotton regulates metabolism, growth, and development by regulating starch synthesis, hydrolysis, and sugar levels.

### Expression and silencing analysis of *GhSS9* under drought stress in cotton

**Figure 8:**
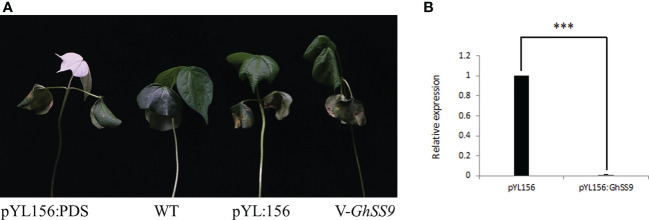
Function verification of *GhSS9*. **(A)** Phenotypic comparison of *GhSS9*-silenced plants under drought stress. **(B)** Detection of *GhSS9* silencing efficiency (***p <0.001).

To understand the potential role of the SS gene family in cotton stress, we selected the *GhSS9* gene for the VIGS experiment. Expression levels of *GhSS9* were significantly reduced in the leaves of *V-GhSS9* plants after VIGS compared with pYL:156 plants (**Figure 8B**), indicating strong and specific silencing of *GhSS9*. To understand the potential role of the SS gene family in cotton stress, we selected the GhSS9 gene for the VIGS experiment. Expression levels of *GhSS9* were significantly reduced in the leaves of *V-GhSS9* plants after VIGS compared with pYL:156 plants (**Figure 8B**), indicating strong and specific silencing of *GhSS9*. After drought stress, the cotyledons of *V-GhSS9* plants turned yellow, the true leaves lost water, and the whole plant seriously wilted. However, the cotyledons of pYL:156 plants showed mild yellowing symptoms, and plant morphology was basically normal after drought stress. *V-GhSS9* plants were more sensitive to drought stress than pYL:156 plants, implying that this gene contributes to drought tolerance in cotton (**Figure 8A**). *V-GhSS9* plants were more sensitive to drought stress than pYL:156 plants, implying that this gene contributes to drought tolerance in cotton (**Figure 8A**).

## Discussion

Sugar is a direct product of plant photosynthesis, transported out of photosynthetic cells in the form of sucrose or stored in the form of starch (Stitt, 1991). Sugar levels (sugar feast or sugar starvation) have a significant impact on plant metabolism and development. As a signaling substance, sugar levels regulate gene expression, metabolic pathways, growth, and development in plants (Gupta and Kaur, 2005). Starch is one of the main polysaccharides in plant cells, which affects the sugar level of plants. Starch synthetase is the key enzyme in starch synthesis in plants. Several SS have been identified in *O. sativa* L. (Zhang et al., 2021), maize (Harn et al., 1998), and wheat (Tan et al., 2010). However, it was still lacking any type of study about SS in cotton. In our study, we identified the SS gene family in *G. arboreum*, *G. raimondii*, *G. hirsutum*, and *G. barbadense*, with the aim of understanding the roles of the SS family in cotton.

Based on the published cotton genomics information (Lin et al., 2010), 76 SS genes were identified by the blastP technique using the SS family genes of *A. thaliana* as target sequences. The proteins encoded by these genes contained 117–1,181 amino acids with a molecular weight of 19.013–135.17 kDa and an isoelectric point of 4.825–8.62 with an average of 6.541, indicating that these proteins were weakly acidic. The SS family has different physical chemistry and functions. By constructing phylogenetic trees of SS genes in *A. thaliana*, *O. sativa*, and cotton, these genes were divided into five branches, and each branch contained SS genes from six species. This means that the SS family differentiated earlier than monocotyledons and dicotyledons. Furthermore, collinearity analysis found fewer repeats between Ga(A)-Ga(A) and Gr(D)-Gr(D), but more repeats between Ga(A)-Gh(AD_1_), Ga(A)-Gb(AD_2_), Gr(D)-Gh (AD_1_), and Gr(D)-Gb(AD_2_). This further verified that *G. hirsutum* (AD_2_) and *G. barbadense* (AD_1_) come from interspecific hybridization between the cotton with the A genome and the cotton with the D genome. During the evolution of cotton, the SS family genes can be preserved, which indicates that the SS family plays an important role in the growth and development of cotton.

The structure of a gene determines its function (Richard et al., 1998). The C-terminal amino acid sequence of SS proteins is highly conserved, but the N-terminal of SS protein is variable, and the conservation is very poor. The genes of the SS family all contain motifs and 4. All SS families contain Glyco_transf_5. We speculate that Glyco_transf_5 may be an important domain of the SS family, mainly composed of motifs 3 and 4. The same subfamily of SS genes has very strong conservation, such as the subfamily III genes, which were composed of motifs 1–9, all of whom contained Glyco_transf_5, Glycos_transf_1, and Glyco_trans_1_4 conserved domains. We hypothesized that different subfamilies of SS have specific roles in starch synthesis.

Three types of response elements were identified in the SS promoter region. The first type was the light response element, with the SS promoter region containing the most cis elements. This was consistent with the conclusion that SS family genes play an important role in starch synthesis during photosynthesis. The second type identified in the SS promoter was the hormone responsiveness element, with 71 SS genes all containing a cis-acting element involved in defense and stress responsiveness (tca-element). Salicylic acid (SA) is a hormone produced by plants and plays an important role in plant growth and stress resistance. It is suggested that the SS gene may be involved in hormone signaling pathways in plants. A third type of stress response element was identified in the SS promoter, suggesting that SS genes may play a key role in response to abiotic stresses.

In our study, we found that some glucose-1-phosphate adenylyltransferase large subunit (APL) genes interact with *GhSS* genes, such as APL1, APL2, APL3, and APL4. Previous studies have shown that the APL family, which encodes the large subunit of the ADP-glucose caramel phosphorylase, catalyzes the first rate-limiting step in starch biosynthesis. GhSSs interact with SBE family genes such as SBE2.1 and SBE2.2. SBE is a key enzyme involved in amylopectin structure formation. Interestingly, both APL and SBE are involved in starch synthesis, which suggests that key enzymes in starch synthesis may interact to promote starch formation (Huang-Lung et al., 2009). Furthermore, *GhSSs* interact not only with starch synthesis-related enzymes but also with starch hydrolysis-related enzymes (DPE1, DPE2) (Ceusters et al., 2019). We hypothesized that *GhSSs* were involved both in starch synthesis and in starch hydrolysis. Starch, as an important storage sugar, can be decomposed to produce soluble sugar under drought stress. The accumulation of soluble sugar under drought stress can be used as an osmotic protective agent to maintain osmotic stability, and it can also act as a ROS scavenger (Krasensky and Jonak, 2012). The rapid gluconeogenic conversion of malate into starch prevents an increase in the volume of the protoplasts, whereas the degradation of starch to malate is accompanied by a swelling of the protoplasts. The mutual transformation of starch and malate constructs the osmotic driving force of stomatal movement, and the gluconeogenesis of malate transforms it into starch. This also supports stomatal closure and mitigates drought stress (Ogawa, 1981; Schnabl, 1980).

**Figure 9:**
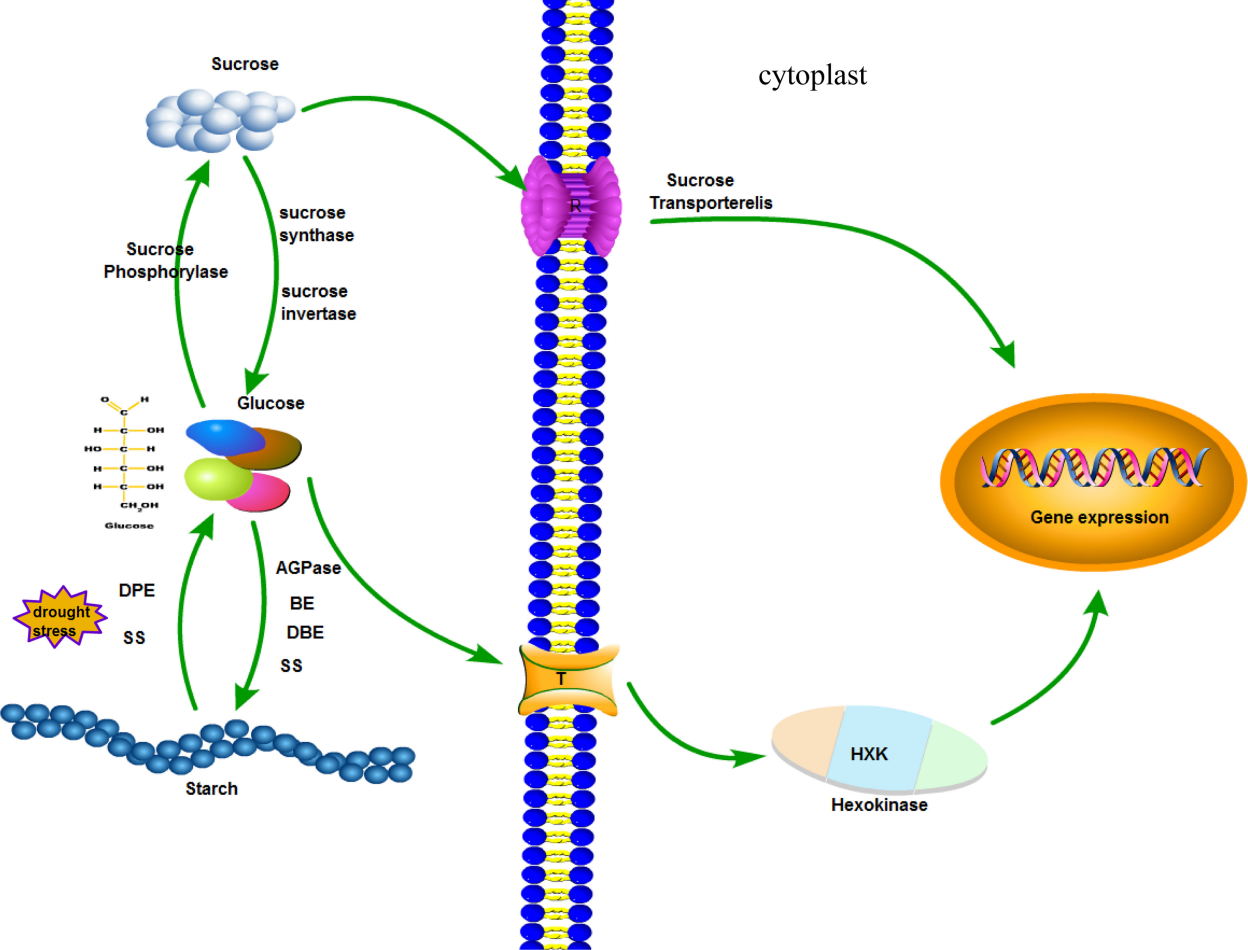
A model for the role of starch synthase (*GhSS*) in the drought resistance of cotton.

The biosynthesis of vascular plant starch takes place in the plastid, and the substrate is sucrose (Ugalde and Jenner, 1990). Sucrose is converted to glucose by sucrose invertase or sucrose synthase (Echeverria and Humphreys, 1984; Moriguchi et al., 1992). Starch is then synthesized by AGPase, SS, SBE, and DBE (Lloyd et al., 1999; Kharabian-Masouleh et al., 2011). There is an interaction between starch synthase and dismutase in the protein interaction network. Starch is degraded to glucose by the actions of dismutase and starch synthetase. Glucose can be converted to sucrose by phosphorylase. The sugar level in the plant has a great influence on the metabolism and growth of the plant (Berger et al., 2010). There are mainly sucrose transporter pathways and glucose receptor pathways (Kühn et al., 2010). The sucrose transporter pathway states that sucrose levels regulate the expression of related genes (Jang et al., 1997; Sheen et al., 1999). As a glucose receptor, hexokinase (HXK) mediates the expression of glucose-related genes (Rolland et al., 2006). When cotton is subjected to drought stress, SS family genes affect the sugar level in the plant by regulating starch synthesis and hydrolysis and regulate gene expression in response to stress (**Figure 9**).

## Conclusion

According to the gene structure, conserved domain, phylogeny, collinearity, chromosome location, and cis-element analysis of the SS family, the characteristics of the SS family in four cotton genomes were studied. In addition, we constructed the gene interaction network of the GhSS protein, and *GhSS9* responds to drought stress by regulating starch synthesis and decomposition. These results lay the foundation for further study of the response of SS genes to abiotic stress.

## Data availability statement

The original contributions presented in the study are included in the article/**Supplementary Material**. Further inquiries can be directed to the corresponding authors.

## Author contributions

QC and ZB designed the project. MD conducted the experiments. XY performed the bioinformatics analysis. MD and XY wrote the manuscript. ZB and QC was responsible for revising the manuscript. All authors contributed to the article and approved the submitted version.

## Supplementary material

Supplementary Table 1 Analysis of Ka/Ks ratios of SS genes in four cotton species.

Supplementary Table 2 Four cotton SS gene collinear gene pairs
